# Enzymes involved in organellar DNA replication in photosynthetic eukaryotes

**DOI:** 10.3389/fpls.2014.00480

**Published:** 2014-09-17

**Authors:** Takashi Moriyama, Naoki Sato

**Affiliations:** ^1^Department of Life Sciences, Graduate School of Arts and Sciences, The University of TokyoTokyo, Japan; ^2^Japan Science and Technology Agency – Core Research for Evolutional Science and TechnologyTokyo, Japan

**Keywords:** plastids, mitochondria, organellar genome, replication

## Abstract

Plastids and mitochondria possess their own genomes. Although the replication mechanisms of these organellar genomes remain unclear in photosynthetic eukaryotes, several organelle-localized enzymes related to genome replication, including DNA polymerase, DNA primase, DNA helicase, DNA topoisomerase, single-stranded DNA maintenance protein, DNA ligase, primer removal enzyme, and several DNA recombination-related enzymes, have been identified. In the reference Eudicot plant *Arabidopsis thaliana*, the replication-related enzymes of plastids and mitochondria are similar because many of them are dual targeted to both organelles, whereas in the red alga *Cyanidioschyzon merolae*, plastids and mitochondria contain different replication machinery components. The enzymes involved in organellar genome replication in green plants and red algae were derived from different origins, including proteobacterial, cyanobacterial, and eukaryotic lineages. In the present review, we summarize the available data for enzymes related to organellar genome replication in green plants and red algae. In addition, based on the type and distribution of replication enzymes in photosynthetic eukaryotes, we discuss the transitional history of replication enzymes in the organelles of plants.

## INTRODUCTION

Plastids and mitochondria are semi-autonomous organelles that contain their own genomes, encoding the genes necessary to perform their respective metabolic functions. These organellar genomes are replicated by specific enzymes, such as DNA polymerase, DNA primase, and DNA helicase, as occurs in bacteria and the nuclei of eukaryotes. In contrast, plant organellar genomes do not encode these replicative proteins, and are instead replicated by nucleus-encoded enzymes that are transported to the organelles. However, the mechanism of plant organellar genome replication is not clearly understood because more than one mode of replication is possible and include recombination-dependent, double D-loop, and rolling circle replication mechanisms (reviewed in Maréchal and Brisson, 2010; Nielsen et al., 2010; Gualberto et al., 2014).

Mitochondria and plastids are thought to have been acquired through endosymbiotic events with ancestors of α-proteobacteria and cyanobacteria, respectively. Studies of bacterial replication enzymes (Langston et al., 2009; Sanyal and Doig, 2012) have shown that homologs of these enzymes also function in plant organelles. In bacterial DNA replication, DnaB helicase unwinds double-stranded DNA (dsDNA) at the replication fork, and the unwound DNA is then prevented from re-annealing with other single-stranded DNAs (ssDNAs) by single-stranded DNA-binding protein (SSB). A type II DNA topoisomerase (gyrase) consisting of A and B subunits alleviates the mechanical strain of unwound DNA. DnaG primase synthesizes an RNA primer, which is elongated by DNA polymerase III and is then removed by nick translation with 5′–3′ exonuclease and the polymerase activity of DNA polymerase I (PolI). The nicked DNA is combined by the NAD^+^-dependent DNA ligase LigA.

All of the enzymes involved in mitochondrial DNA replication in vertebrates have been identified (Arnold et al., 2012; Kasiviswanathan et al., 2012). DNA polymerase γ (Polγ) functions in the replication and repair of animal mitochondrial DNA. Animal Polγ consists of two subunits: a large subunit with DNA polymerase and 3′–5′ exonuclease activities, and a small subunit that functions in primer recognition and enhances processivity (Kaguni, 2004; Wanrooij and Falkenberg, 2010). The animal mitochondrial primase, POLRMT, which has homology to the RNA polymerase of T3/T7 phage, was recently indicated to function in both primer synthesis and transcription, although it was previously thought to function only in transcription (Wanrooij et al., 2008). Plants also have homolog(s) of T3/T7 phage RNA polymerase, which are named RPOTs (RNA polymerase of the T3/T7 type) and are localized to plastids and/or mitochondria, where they function in transcription (Kühn et al., 2007). Mitochondrial helicase is called TWINKLE and has homology to the gp protein of T7 phage, which contains primase and helicase domains at the *N*- and *C*-termini, respectively. However, although the TWINKLE found in bikonts (plants and protists) has both primase and helicase activities, animal TWINKLE only shows helicase activity (Shutt and Gray, 2006). A number of other replication-related enzymes, including topoisomerases 1 and 3a, SSB, ligase 3, and RNase H1, have also been identified in human mitochondria. An *in vitro* reconstituted mitochondrial replisome composed of Polγ, TWINKLE, and SSB displayed rolling-circle replication with high processivity (Korhonen et al., 2004).

Several of the replicative enzymes found in bacteria and animal mitochondria are also encoded by plant nuclear genomes. In addition to these common enzymes, a number of plant-specific enzymes for DNA replication and recombination have recently been identified, and their subcellular localization has been examined in both plants and algae. In this article, we summarize the current knowledge on enzymes related to organellar replication in photosynthetic eukaryotes and also discuss the evolution of these replication-related enzymes based on their distribution in photosynthetic eukaryotes.

## REPLICATION DNA POLYMERASE, POP

### HISTORY OF STUDIES ON ORGANELLAR DNA POLYMERASES IN PLANTS

**FIGURE 1:**
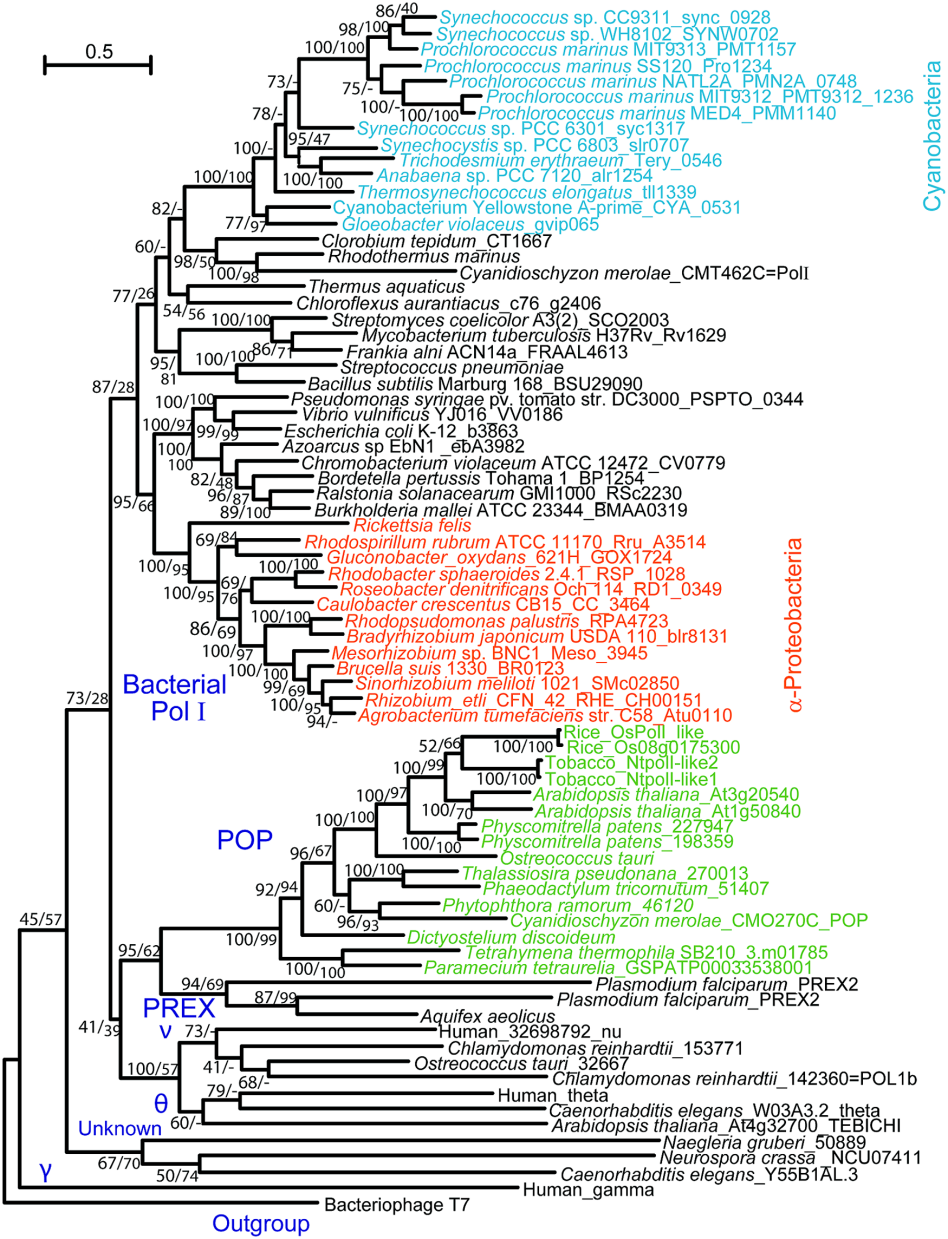
**Phylogenetic tree of Family A DNA polymerases.** Reproduced from Moriyama et al. (2014) with permission.

DNA replication activity was first detected in isolated organelles from plants, yeasts, and animals in the late 1960s (Wintersberger, 1966; Parsons and Simpson, 1967; Spencer and Whitfeld, 1967; Tewari and Wildman, 1967). In the following decade, DNA polymerases were purified from isolated chloroplasts and mitochondria of various photosynthetic eukaryotes (summarized in Moriyama and Sato, 2013). Biochemical data suggested that plant organellar and γ-type DNA polymerases, which are responsible for replication of the mitochondrial genome in fungi and animals (Lecrenier and Foury, 2000; Kaguni, 2004), had similar optimal enzymatic conditions, particularly pH and the concentration of monovalent and divalent ions, sensitivity to DNA polymerase inhibitors, molecular size, and template preference. Despite such biochemical evidence, no gene encoding a homolog of Polγ has been found in the sequenced genomes of bikonts, including those of plants and protists, and the organellar DNA polymerase in photosynthetic organisms remains unidentified. Sakai et al. (1999) and Sakai (2001) detected DNA synthetic activity in the nucleoid fraction isolated from chloroplasts and mitochondria of tobacco and determined that the apparent molecular mass of the enzyme exhibiting the activity was similar to the Klenow fragment of PolI in *Escherichia coli*. This finding led to the identification of a gene(s) encoding a DNA polymerase with distant homology to *E. coli* PolI in the genomes of bikonts. The identified DNA polymerase was first isolated from plastids of rice, and its localization was confirmed by immunoblot analysis of isolated plastids (Kimura et al., 2002). Subsequent studies using GFP-fusion proteins and/or immunoblotting demonstrated that the polymerases, which were named PolI-like, PolI or Polγ, are localized to both plastids and mitochondria in *Arabidopsis thaliana* and tobacco (Christensen et al., 2005; Mori et al., 2005; Ono et al., 2007; Parent et al., 2011; Cupp and Nielsen, 2013). We also identified this type of DNA polymerase in algae and ciliates (Moriyama et al., 2008, 2011, 2014). In these reports, phylogenetic analysis of Family A DNA polymerases revealed that plant organellar DNA polymerases belong to a clade that is distinct from that of bacterial PolI and Polγ (**Figure 1**). In addition, red algae were found to encode a DNA polymerase with high homology to *E. coli* PolI (Moriyama et al., 2008). Therefore, we proposed that this type of organellar DNA polymerase be named POP (plant and protist organellar DNA polymerase), because the genes encoding the polymerases are present in both photosynthetic eukaryotes and protists.

### ENZYMATIC CHARACTERISTICS OF POPs

#### Characterization of DNA polymerase activity of POPs

The DNA polymerase activity of POPs has been characterized using recombinant (Kimura et al., 2002; Ono et al., 2007; Takeuchi et al., 2007) and native proteins purified from *Cyanidioschyzon merolae* (red alga) and isolated mitochondria of *Tetrahymena thermophila* (Moriyama et al., 2008, 2011). The optimal KCl concentration for POP polymerase activity is 50–150 mM. POPs also show divalent metal ion-dependent activity, and the optimal MgCl_2_ concentration for their activity is 2.5–5 mM. POPs exhibit the highest activity with poly(dA)/oligo(dT) as a template, rather than with activated calf thymus DNA. However, poly(rA)/oligo(dT) can also be used as a template, indicating that POPs have reverse transcriptase activity, similar to Polγ, although the *in vivo* role of this activity has not been elucidated.

**FIGURE 2:**
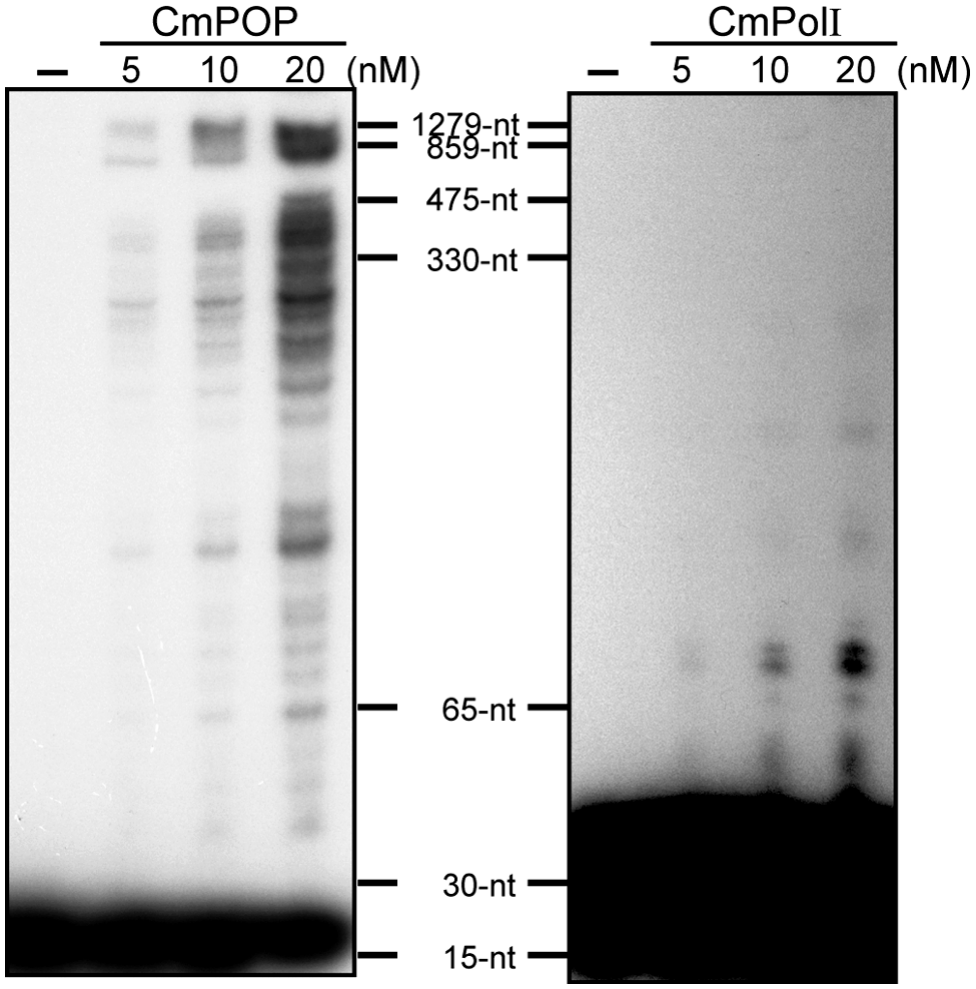
**Processivity of CmPOP and CmPolI.** Reactions were performed in reaction buffer (50 mM Tris-HCl [pH 8.0], 5 mM MgCl_2_, 1 mM DTT, 0.01% BSA, 50 μM dATP, 50 μM dTTP, 50 μM dGTP, 50 μM dCTP, and 10 nM ^32^P–labeled M13mp18-M1) at 37°C for 5 min. The reaction products were analyzed in 5% polyacrylamide gels containing 7 M urea. Reproduced from Moriyama et al. (2008) with permission.

DNA polymerase enzymes bind to and dissociate from template DNA repeatedly during the replication or repair process. The number of synthesized nucleotides added by the DNA polymerase per one binding event is defined as processivity. POPs show markedly high processivity values, ranging from 600 to 900 nt for recombinant rice POP and 1,300 nt for *Cyanidioschyzon merolae* POP (**Figure 2**, Moriyama et al., 2008). In comparison, *E. coli* PolI has mid-range processivity of <15 nt (Takeuchi et al., 2007). The *Cyanidioschyzon merolae* genome encodes a *PolI* gene (*CmPolI*) having high homology with *E. coli* PolI, and CmPolI also has mid-range processivity of <70 nt (Moriyama et al., 2008). Alignment analysis of POPs with other Family A DNA polymerases revealed that POP proteins have additional sequences that are involved in DNA binding and synthesis activities, suggesting that the high processivity of POPs might be attributable to these extra sequences (Takeuchi et al., 2007). In animals, an accessory subunit (PolγB) of Polγ enhances the processivity of PolγA. For example, the processivities of the *Drosophila* PolγA subunit and a Polγ holoenzyme consisting of PolγA and PolγB are <40 nt and >1,000 nt, respectively (Williams et al., 1993). In contrast, POPs show high processivity as a single subunit enzyme, and to our knowledge, no accessory proteins associated with POP have been identified.

#### Sensitivity to DNA polymerase inhibitors

The effect of inhibitors, such as aphidicolin, *N*-ethylmaleimide (NEM), dideoxyTTP (ddTTP), and phosphonoacetate (PAA), on the DNA synthesis activity of POPs was evaluated (Kimura et al., 2002; Ono et al., 2007; Moriyama et al., 2008, 2011). Aphidicolin is an inhibitor of eukaryotic nuclear DNA polymerases α, δ, and 𝜀 (Holmes, 1981; Weiser et al., 1991), and did not inhibit POP activity (Moriyama et al., 2008). NEM is an inhibitor of DNA polymerases α, γ, δ, and 𝜀 (Chavalitshewinkoon-Petmitr et al., 2000), and also did not inhibit POP. ddTTP, which severely impairs the activity of DNA polymerases β and γ (Kornberg and Baker, 1992), had various inhibitory effects on POP depending on the organism, with the half maximal inhibitory concentrations (IC_50_) ranging from 4 to 615 μM for the POPs of *A. thaliana*, *Cyanidioschyzon merolae*, and the ciliate *Tetrahymena* (Moriyama et al., 2011). PAA was originally identified as an inhibitor of viral DNA polymerases and reverse transcriptases, and functions by interacting with pyrophosphate binding sites, leading to an alternative reaction pathway (Leinbach et al., 1976; Shiraki et al., 1989). PAA severely inhibits the activity of POPs, with IC_50_ values of 1–25 μM (Moriyama et al., 2011). Because PAA at low concentrations does not inhibit other Family A DNA polymerases, such as PolI and Polγ, sensitivity to PAA is a useful marker for the classification of organelle-localized DNA polymerases in unsequenced eukaryotes (Moriyama et al., 2011).

#### 3′–5′ exonuclease activity

Plant and protist organellar DNA polymerases have a 3′–5′ exonuclease domain at the *N*-terminus consisting of three conserved regions, Exo I, Exo II, and Exo III. This exonuclease activity was shown in rice (Takeuchi et al., 2007) and *Cyanidioschyzon* (Moriyama et al., 2008). In rice POP, a mutant protein containing a replacement of Asp365 with Ala in the Exo II domain lost 3′–5′ exonuclease activity. In terms of 3′–5′ exonuclease proofreading activity, rice POP exhibited a relatively high fidelity at a base substitution rate of 10^-4^ to 10^-5^ (Takeuchi et al., 2007).

### EXPRESSION OF POP IN PLANTS

In tobacco BY-2 cells, the amount of POP transcripts and proteins increases at the initial phase of plastidial and mitochondrial DNA replication (Ono et al., 2007). The spatial expression patterns of POPs were analyzed in *A. thaliana* and rice by *in situ* hybridization, which revealed that POP genes are strongly expressed in the apical meristems of roots and shoots, leading to high POP protein levels in these tissues (Kimura et al., 2002; Mori et al., 2005). In *A. thaliana*, the expression of two POPs, AtPOP1 (At1g80840) and AtPOP2 (At3g20540), were compared by quantitative RT-PCR (Cupp and Nielsen, 2013). The analysis demonstrated that AtPOP1 is mainly expressed in rosette leaves, whereas AtPOP2 is predominantly found in the meristems of roots and shoots.

### EXPRESSION OF POP IN ALGAE

The unicellular red alga *Cyanidioschyzon merolae* contains a single plastid and mitochondrion (Matsuzaki et al., 2004), whose division cycles are synchronized with the cell cycle. In synchronous cultures of *Cyanidioschyzon merolae* established using light–dark cycles (Suzuki et al., 1994), cells divide at ∼12 h from the beginning of the light phase, and nuclear DNA increases at or just before the M-phase. Replication of the mitochondrial genome appears to be at least partially synchronized with the cell cycle, as mitochondrial DNA begins to replicate from the light phase, and reaches a two-fold increase at or near the M-phase. In contrast, plastid DNA increases gradually throughout the entire cell cycle, even after cell division is complete (Moriyama et al., 2010). In contrast to land plants, which typically encode two or more copies of POP, *Cyanidioschyzon merolae* only has a single POP. The mRNA level of *CmPOP* changes during the cell cycle and reaches a peak that correlates with the rise in the mitotic index (Moriyama et al., 2008). However, the protein level of POP remains nearly unchanged throughout the cell cycle, with only small increases and decreases occurring during the light and dark phases, respectively. The observed expression of CmPOP is consistent with the results of organellar DNA replication during the cell cycle of *Cyanidioschyzon merolae*.

### PHENOTYPES OF POP MUTANTS IN PLANTS

*POP* mutants of *A. thaliana* have been characterized by two research groups (Parent et al., 2011; Cupp and Nielsen, 2013). The *A. thaliana* genome encodes two POPs, AtPOP1 and AtPOP2, which are both localized to plastids and mitochondria. Double mutation of AtPOP1 and AtPOP2 was lethal, whereas each single mutant showed reduced DNA levels in both plastids and mitochondria. Additionally, the *Atpop2* mutant displayed high sensitivity to ciprofloxacin, an inducer of DNA double-strand breaks. These results indicate that two distinct POPs are involved in genome replication for plastids and mitochondria, and that AtPOP2 also functions in DNA repair in both organelles.

## OTHER REPLICATION ENZYMES OF ORGANELLAR GENOMES

### DNA PRIMASE AND HELICASE

DNA helicase unwinds dsDNA to allow DNA replication by DNA polymerase. In *E. coli*, primase synthesizes an RNA primer at the origin of replication in the leading strand and every ∼1 kb in the lagging strand. TWINKLE (T7 gp4-like protein with intramitochondrial nucleoid localization), which is a homolog of the T7 phage gp4 protein with primase and helicase activities, was originally reported to function as a hexameric DNA helicase in human mitochondria (Spelbrink et al., 2001). In *A. thaliana*, TWINKLE functions as a DNA helicase and primase (Diray-Arce et al., 2013), and was shown to be localized to both chloroplasts and mitochondria by GFP-tagging experiments (Carrie et al., 2009). Dual-targeted enzymes to the mitochondria and chloroplasts of plants are summarized in the review by Carrie and Small (2013). In an assay using single-stranded M13 DNA as a template, a recombinant TWINKLE protein of *A. thaliana* (AtTWINKLE) showed ATP-dependent helicase and primase activities, synthesizing RNA primers of >15 nt that were then extended by *E. coli* PolI into high-molecular-weight DNA (Diray-Arce et al., 2013). The protein and mRNA of AtTWINKLE are mainly expressed in the meristem and young leaves, which is similar to the expression pattern of *A. thaliana* POPs, particularly *AT3G20540*. The *A. thaliana* genome encodes a second *TWINKLE* gene whose protein product only has the *N*-terminal primase domain of TWINKLE and is localized to chloroplasts, according to unpublished data in the review by Cupp and Nielsen (2014).

Red algae also have a TWINKLE protein; however, it is localized to only mitochondria (Moriyama et al., 2014). Red algae and diatoms have a plastid-encoded DnaB helicase and a nucleus-encoded DnaG primase. In our analysis using GFP in *Cyanidioschyzon merolae*, DnaG was localized to the plastid. We also confirmed the plastid-localization of DnaG in the red alga *Porphyridium purpureum*. Based on these data, it appears that red algae and diatoms, the latter of which is thought to have originated from the secondary endosymbiosis with a red alga, utilize DnaB/DnaG in plastids and TWINKLE in mitochondria.

### DNA TOPOISOMERASE

*Arabidopsis thaliana* has a single gyrase A (AtGYRA) that is localized to both plastids and mitochondria, and has two gyrase B enzymes that are localized to either chloroplasts (AtGYRB1) or mitochondria (AtGYRB2; Wall et al., 2004). T-DNA insertion mutation of *AtGYRA* leads to an embryo-lethal phenotype, whereas T-DNA insertion mutations of both plastidial *AtGYRB1* and mitochondrial *AtGYRB2* result in seedling-lethal phenotypes (Wall et al., 2004). In *Nicotiana benthamiana*, virus-induced silencing of the genes encoding GYRA and GYRB resulted in abnormal nucleoid content and structure of chloroplasts and mitochondria (Cho et al., 2004). *A. thaliana* also encodes a gyrase B-like gene, *GYRB3*; however, it is recently reported that *AtGYRB3* does not encode a gyrase subunit, as AtGYRB3 showed no supercoiling activity and did not interact with AtGYRA (Evans-Roberts et al., 2010). In addition to gyrases, plant organelles contain A-type topoisomerase I, which is a homolog of bacterial topoisomerase I (TopA). Based on localization analysis using a GFP-fusion protein, AtTOP1 was shown to be localized to both chloroplasts and mitochondria (Carrie et al., 2009).

The genome of *Cyanidioschyzon merolae* encodes genes for GYRA and GYRB, which are localized only to the plastid (Moriyama et al., 2014). Similarly, *Cyanidioschyzon merolae* TOP1 (type IA) is also localized only to the plastid. To search for mitochondrial topoisomerases, we examined the subcellular localization of topoisomerases encoded in the *Cyanidioschyzon merolae* genome, and showed that a homolog of eukaryotic TOP2 is targeted to mitochondria. To date, organellar localization of eukaryotic TOP2 has not been reported in plants. In *Cyanidioschyzon merolae*, the gyrase specific inhibitor nalidixic acid arrests not only replication of the plastid genome, but also that of the mitochondrial and nuclear genomes (Itoh et al., 1997; Kobayashi et al., 2009). The localization results of gyrases in *Cyanidioschyzon merolae* suggest that defective plastid replication leads to the arrest of mitochondrial and nuclear replication by a yet unknown mechanism.

### DNA LIGASE

DNA ligase is required for DNA replication, repair, and recombination, as it seals nicked-DNA ends of single-stranded breaks or joins DNA ends after double-stranded breaks. Four DNA ligases have been identified in the *A. thaliana* genome. *A. thaliana* DNA ligase 1 (AtLIG1) is targeted to either the mitochondria or the nucleus when the gene transcript is translated from the first and second initiation codons, respectively (Sunderland et al., 2004, 2006). AtLIG1 is expressed in all tissues of *A. thaliana*, but higher transcript levels are found in young leaves and tissues containing meristem (Taylor et al., 1998). Plastid-targeting of AtLIG1 was not observed for any AtLIG1-GFP constructs translated from possible initiation codons, and the plastidial enzyme functioning as DNA ligase remains unclear. However, it has been noted that AtLIG6 has a putative plastid-targeting peptide at the *N*-terminus and might therefore be targeted to plastids (Sunderland et al., 2006).

*Cyanidioschyzon merolae* has a single gene encoding DNA ligase. *Cyanidioschyzon merolae* DNA ligase 1 (CmLIG1) has two methionine residues in its *N*-terminal region and is targeted to both mitochondria and plastids when the transcript is translated from the first and second initiation codons (Moriyama et al., 2014). In our analysis, no nuclear localization was observed when the *N*-terminal peptide of CmLIG1 was fused with GFP. However, the protein subcellular localization prediction software WolfPSORT () detected a nuclear localization signal in CmLIG1. Therefore, CmLIG1 appears to have triple localization in plastids, mitochondria, and the nucleus.

### SINGLE-STRANDED DNA (ssDNA)-BINDING PROTEIN

An SSB, AtSSB1, was identified in *A. thaliana* (Edmondson et al., 2005). AtSSB1 is localized to mitochondria, but was also reported to be localized to chloroplasts in the review by Cupp and Nielsen (2014). AtSSB1 binds to ssDNA, but not to dsDNA, and stimulates RecA-mediated strand exchange activity.

Organellar ssDNA-binding proteins (OSBs) comprise the second class of SSBs in plants (Zaegel et al., 2006). OSBs have an SSB-like domain in the central region and one, two, or three C-terminal PDF motifs, which consist of 50-amino acids and are responsible for ssDNA binding. PDF motifs are conserved only in green plants, including *Chlamydomonas reinhardtii*. *A. thaliana* has four OSBs: AtOSB1 and 2 are localized to mitochondria and chloroplasts, respectively, whereas AtOSB3 is localized to both chloroplasts and mitochondria. AtOSB1 and AtOSB2 have been purified as recombinant proteins that showed preferential binding activity to ssDNA, as compared to dsDNA or RNA. Expression analysis of *AtOSB1* using a β-glucuronidase (GUS) assay demonstrated that *AtOSB1* is mainly expressed in gametophytic cells. T-DNA insertion mutation of the *OSB1*, *OSB2*, and *OSB3* genes revealed that *osb1* mutants accumulate homologous recombination products of mitochondrial DNA, whereas *osb2* and *osb3* mutants have no visible phenotype. These findings, together with the expression analysis for *AtOSB1*, indicate that AtOSB1 is involved in mitochondrial DNA recombination in gametophytic cells (Zaegel et al., 2006).

Replication protein A (RPA) is a nucleus-localized BBC in eukaryotes and is comprised of three subunits, RPA70, RPA32, and RPA14. The rice genome encodes three RPA70s, three RPA32s, and one RPA14. These RPA subunits combine in different variations to make three types of complexes: type A, B, and C. Among these RPA complexes, the type A complex is localized to chloroplasts in rice (Ishibashi et al., 2006).

The *Cyanidioschyzon merolae* genome encodes a single gene for SSB, but does not contain a gene for OSB. In our analysis, the SSB of *Cyanidioschyzon merolae* is localized only in the mitochondrion, unlike that of *A. thaliana* (Moriyama et al., 2014). We performed the localization analysis using a construct starting from the second methionine codon or starting from the ATA codon located upstream of the first methionine codon; however, none of the constructs showed plastid localization. We also examined the organellar localization of RPAs in *Cyanidioschyzon merolae*, and even though they have no extension sequence at the *N*-terminus, they were localized to the nucleus. Based on these findings, the plastidial SSB in red algae remains unidentified.

### PRIMER REMOVAL ENZYME

In *E. coli*, RNA primers are removed by nick translation with the 5′–3′ exonuclease and polymerase activities of DNA polymerase I (Langston et al., 2009; Sanyal and Doig, 2012). In contrast, RNaseH1 performs this role in human mitochondria (Kasiviswanathan et al., 2012). Although there are no reports of RNA primer removal enzymes that are specific to the organelles in green plants, two 5′–3′ exonucleases (5′–3′EXO1 and 2) having sequence homology to the 5′–3′ exonuclease domain of *E. coli* PolI are predicted to be localized to chloroplasts or mitochondria (Sato et al., 2003).

*Cyanidioschyzon merolae* has a gene with high sequence homology to bacterial PolI (Moriyama et al., 2008). The corresponding protein, CmPolI, contains 5′–3′ exonuclease and polymerase domains. We demonstrated the plastid localization of CmPolI by immunoblotting and observation of a CmPolI-GFP fusion protein (Moriyama et al., 2008, 2014). Because CmPolI has low processive polymerase activity and no 3′–5′ exonuclease activity, the enzyme appears to function in repair and primer removal by nick translation, similar to PolI in *E. coli*. We also showed that *Cyanidioschyzon merolae* RNase HII is localized to the mitochondrion, and that DNA2 nuclease/helicase and FEN1 are localized to the nucleus (Moriyama et al., 2014).

## PHYLOGENETIC DISTRIBUTION OF ORGANELLAR REPLICATIVE ENZYMES

### ORIGIN OF ENZYMES RELATED TO ORGANELLAR GENOME REPLICATION

**FIGURE 3:**
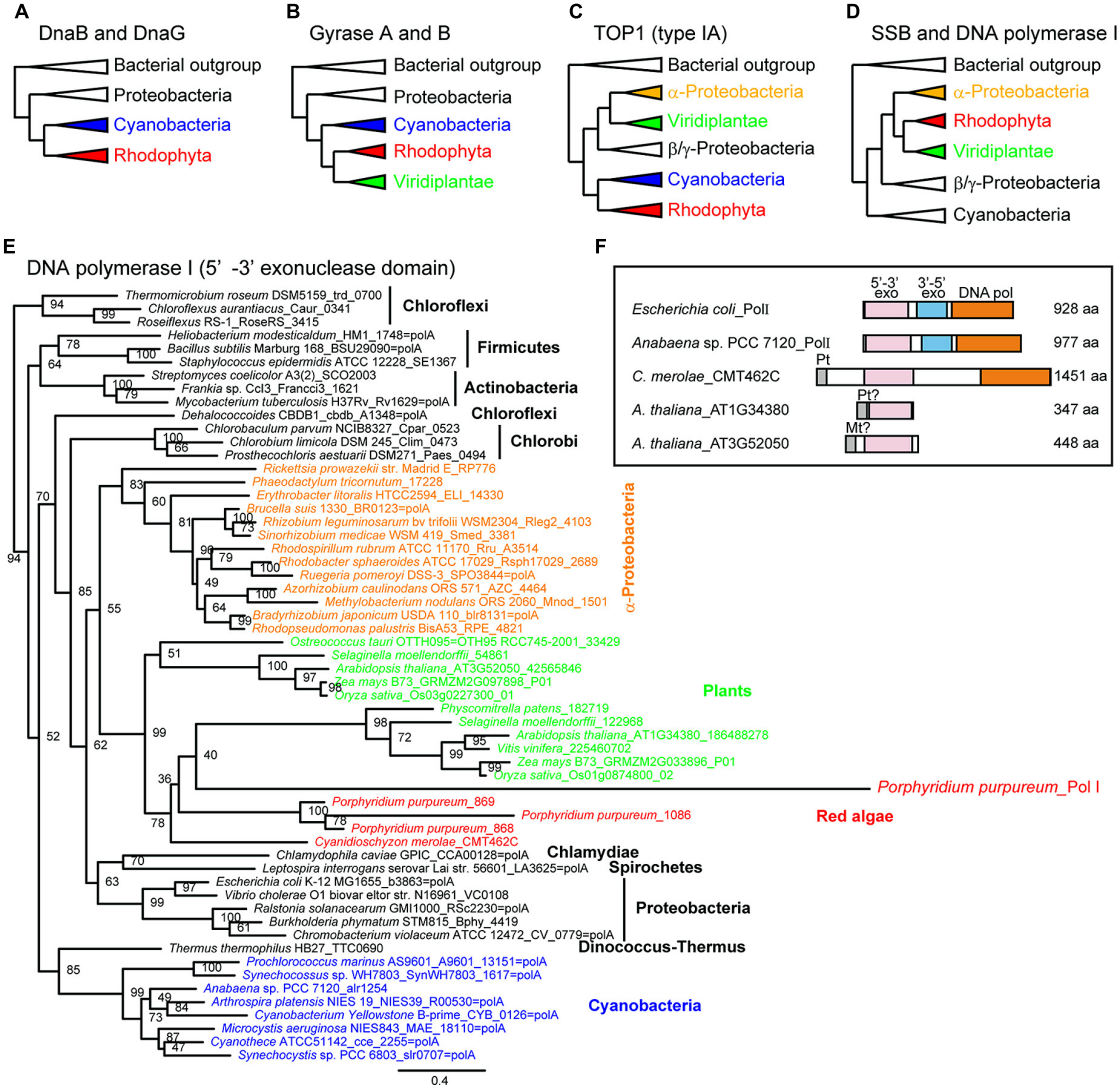
**Phylogenetic trees of enzymes related to organellar genome replication.** Simplified phylogenetic trees **(A–D)**. A detailed phylogenetic tree based on plant 5′–3′ exonuclease and the 5′–3′ exonuclease domains of bacterial PolI **(E)**. Schematic comparison of the structure of PolI and plant 5′–3′ exonuclease **(F)**. Modified from Moriyama et al. (2014) with permission.

Phylogenetic analyses of bacterial-type replicative enzymes have been performed (Moriyama et al., 2014). We previously suggested that POP did not originate from the PolI of α-proteobacteria or cyanobacteria (**Figure 1**, Moriyama et al., 2008). However, the analyses indicated that red algal DnaB helicase and DnaG primase originated from cyanobacteria (**Figure 3A**). Gyrases A and B also originated from cyanobacteria in both green plants and red algae (**Figure 3B**). Type IA topoisomerase was derived from α-proteobacteria in green plants and from cyanobacteria in red algae (**Figure 3C**). SSB and 5′–3′ exonuclease/PolI originated from α-proteobacteria in green plants and red algae (**Figure 3D**). With regard to PolI, as green plants have enzymes with 5′–3′ exonuclease domain, but lack 3′–5′ exonuclease and DNA polymerase domains (**Figure 3F**), phylogenetic analysis of the 5′–3′ exonuclease domain in bacteria and photosynthetic eukaryotes was performed (**Figures 3D,E**). These results suggest that the organelle replication apparatus of both green plants and red algae is composed of enzymes of various origins, including α-proteobacteria, cyanobacteria, and eukaryotes.

### REPERTOIRE OF ENZYMES RELATED TO ORGANELLAR GENOME REPLICATION IN PLANTS AND ALGAE

The enzymes related to organellar DNA replication and recombination in a species of angiosperm, fern, moss, filamentous terrestrial alga, two green algae, and two red algae are listed in **Table 1**. The proteins conserved in the all examined species are POP, TWINKLE, Gyrases, type IA-TOP1, TOP2, and LIG1. DnaB and DnaG are conserved only in red algae. The retention of SSBs is highly variable in photosynthetic eukaryotes. Bacterial-type SSB proteins are conserved in land plants and *Cyanidioschyzon merolae*, whereas OSB proteins are conserved among land plants, including *A. thaliana*, *Physcomitrella patens*, and *Klebsormidium flaccidum*. It was reported that OSB proteins contain a few PDF motifs in addition to an SSB-like domain (Zaegel et al., 2006). According to this classification, *Physcomitrella patens* and *K. flaccidum* have a single OSB, which contains one and two PDF motifs, respectively, in addition to the SSB-like domain. RECA and Whirly (WHY) are recombination-related proteins and are also not uniformly conserved in photosynthetic eukaryotes. For example, *Selaginella moellendorffii* and *Porphyridium purpureum* do not have RECA, red algae do not encode WHY, and *Physcomitrella patens* has neither RECA nor WHY. Conservation of origin-binding protein (ODB) is more limited, as only land plants have this protein. PolI containing 5′–3′ exonuclease and DNA polymerase domains is retained only in red algae. However, a protein with the 5′–3′ exonuclease domain is found in photosynthetic eukaryotes, with the exception of *Cyanidioschyzon merolae*. Therefore, all photosynthetic eukaryotes contain proteobacteria-derived PolI. RNase H having high homology to RNase HII of *Cyanidioschyzon merolae* is conserved in most plants and algae, with the notable exception of *A. thaliana*. The observed distribution of enzymes that play key roles in replication indicates that they are essentially conserved in all plants and algae. In contrast, because recombination-related enzymes and SSBs are non-uniformly distributed among plants and algae, these enzymes are considered to exhibit high plasticity during evolution.

**Table 1:** List of replication-related enzymes possibly localized to plastids or mitochondria in photosynthetic eukaryotes.

		Angiospermae	Pteridophyta	Bryophyta	Charophyta	Chlorophyta		Rhodophyta	
Function	Protein name	*Arabidopsis thaliana*	*Selaginella moellendorffii*	*Physcomitrella patens*	*Klebsormidium flaccidum*	*Chlamydomonas reinhardtii*	*Ostreococcus lucimarinus*	*Cyanidioschyzon merolae*	*Porphyridium purpureum*
DNA polymerase	POP	AT1G50840 (**pt, mt**)	82310^a^	008G080400 (pt, mt)	kfl00710_0020 (pt, mt)	g8521 (pt, mt)	34610^a^	CMO270C (**pt, mt**)	contig2347.14 (pt, mt)
		AT3G20540 (**pt, mt**)		014G054900 (pt, mt)					
				024G060900					
Primase/helicase	TWINKLE	AT1G30680 (**pt, mt**)	164801^a^	012G092800^a^	kfl00614_0010 (pt, mt)	Cre12.g503150 (pt)	94562^a^	CMT452C (**mt**)	contig653.2 (partial)
		AT1G30660 (primase domain) (**pt**)				Cre12.g514950 (pt, mt)			contig653.4 (partial)
Primase	DnaG	N.D.	N.D.	N.D.	N.D.	N.D.	N.D.	CMQ286C (**pt**)	contig2059.10 (pt)
Helicase	DnaB	N.D.	N.D.	N.D.	N.D.	N.D.	N.D.	CMV098C (encoded by ptDNA)	contig622.1 (encoded by ptDNA)
Topoisomerase	GYRA	AT3G10690 (**pt, mt**)	111960 (pt)	008G071000 (pt, mt)	kfl00194_0180 (pt, mt)	Cre04.g221650 (pt)	28121 (pt, mt)	CMS243C (**pt**)	contig445.3 (pt)
	GYRB	AT3G10270 (**pt**)	405784 (pt, mt)	019G017500	kfl00564_0010 (mt)	Cre10.g440750 (pt, mt)	30508 (pt, mt)	CMH166C (**pt**)	contig2132.10 (pt)
		AT5G04130 (**mt**)		022G014300 (pt)					
	TOP1 (type IA)	AT4G31210 (**pt, mt**)	123748^a^	023G038200^a^	kfl00696_0070 (pt, mt)	Cre10.g442850 (pt, mt)	41359 (pt, mt)	CMI252C (**pt**)	contig3627.1 (pt)
				024G035000^a^					
	TOP2	AT3G23890 (pt, nuc)	98330 (nuc)	018G068300 (nuc)	kfl00053_0310 (nuc)	Cre01.g009250 (nuc)	41836 (nuc)	CMB013C (**nuc**)	contig2019.13 (nuc)
			127267 (nuc)	019G011100 (nuc)	kfl00219_0040 (pt, mt)			CML330C (**mt**)	contig3477.2 (mt)
					kfl00568_0100 (pt)				
					kfl00586_0110p^a^				
					kfl00728_0040 (pt, mt)				
					kfl01072_0010 (pt)				
					kfl01385_0010 (pt)				
ssDNA binding protein	SSB	AT3G18580 (mt)	115961^a^	005G033200 (mt, nuc)	N.D.	N.D.	10587^a^	CMI135C (**mt**)	N.D. [-13pt]
	AT4G11060 (**pt, mt**)	440287 (pt, mt)	006G0532001^a^					
				016G055400 (pt, mt)					
	OSB	AT1G31010 (**mt**)	N.D.	021G006400 (pt, mt)	kfl00106_0250 (pt, mt)	N.D.	N.D.	N.D.	N.D.
		AT1G47720 (**mt**)							
		AT4G20010 (**pt**)						
		AT5G44785 (**pt, mt**)							
Ligase	LIG1	AT1G08130 (**mt, nuc**)	96808^a^	013G068900 (pt)	kfl00242_0010 (none)	g7530 (nuc)	16988 (none)	CMK235C (**pt, mt**)	contig730.1 (pt, mt)
		AT1G49250 (pt, nuc)	97073^a^		kfl00698_0030 (none)	g6320 (pt, mt)			
Primer removal	5′–3′ EXO	AT1G34380 (pt)	46231^a^	001G092400 (pt)	kfl00249_0020^a^	g4419^a^	5068^a^	N.D.	contig3646.3 (pt, mt)
	AT3G52050 (mt)	46232^a^	016G072100 (pt, mt)	kfl00340_0150^a^				contig3646.4 (pt, mt)
		54861^a^						
			105035^a^						
			416415 (pt)						
	PolI (5′–3′EXO … Pol)	N.D.	N.D.	N.D.	N.D.	N.D.	N.D.	CMT462C (**pt**)	contig2156.11 (nuc)
	RNase HII^b^	N.D.	91138^a^	002G083400 (pt, mt)	kfl00183_0070 (pt, mt)	Cre13.g561900 (pt, mt)	7887^a^	CMT626C (**mt**)	contig4525.1 (pt, mt)
Recombinase	RECA	AT1G79050 (**pt**)	N.D.	N.D.	kfl00358_0070 (pt, mt)	Cre07.g314650 (pt)	26240^a^	CMK061C (pt, mt)	N.D.
	AT2G19490 (**pt, mt**)			kfl00493_0080 (pt, mt)				
		AT3G10140 (**mt**)							
		AT3G32920 (**mt**)							
Recombination mediator	WHY	AT1G14410 (**pt**)	84708 (nuc)	N.D.	kfl00637_0100 (pt, mt)	Cre02.g091550 (pt, mt)	8400^a^	N.D.	N.D.
	AT1G71260 (**mt**)							
		AT2G02740 (**pt**)							
	ODB	AT1G71310 (**mt, nuc**)	68768^a^	001G130700 (pt)	kfl00130_0080^a^	N.D.	N.D.	N.D.	N.D.
		AT5G47870 (**pt, nuc**)		015G028700 (pt, nuc)	(pt, mt, nuc)				

*Enzymes were estimated by BLAST searches at the websites of Phytozome v9.1 (www.phytozome.net/), *Klebsormidium flaccidum* Genome Project (www.plantmorphogenesis.bio.titech.ac.jp/~algae_genome_project/klebsormidium/index.html), and *Porphyridium purpureum* genome project (cyanophora.rutgers.edu/porphyridium/), and protein IDs are shown. The results of subcellular localization reported in *A. thaliana* and *Cyanidioschyzon* are indicated by pt (plastids), mt (mitochondria), or nuc (nucleus) in bold in parentheses, and references are indicated in the body text. Localization of enzymes in other species was estimated by the subcellular-targeting estimation programs TargetP (www.cbs.dtu.dk/services/TargetP/) and WolfPSORT (wolfpsort.org/)*

*^a^The obtained protein sequence did not appear to encode for a full-length protein containing an initiation methionine residue; ^b^RNase HII with high homology to C. merolae protein (CMT626C); N.D., not detected.*

**FIGURE 4:**
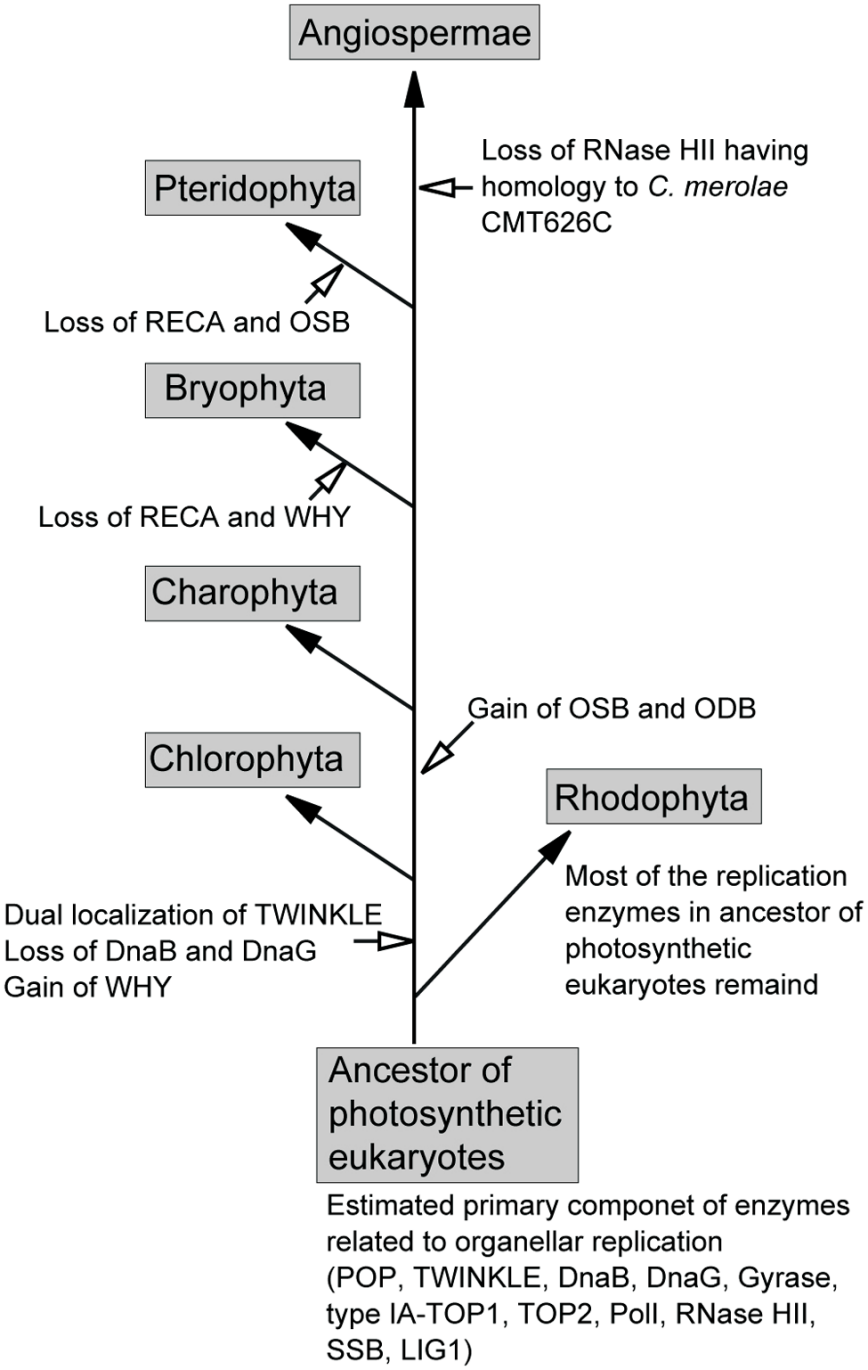
**Proposed model for the exchange of organellar replication enzymes during the evolution of photosynthetic eukaryotes**.

Based on the presence of enzymes related to organellar genome replication in plant genomes, we propose a model for the substitution of these enzymes in photosynthetic eukaryotes (**Figure 4**). In this model, the ancestor of photosynthetic eukaryotes, prior to its divergence into green and red lineages, contained POP, TWINKLE, DnaB, DnaG, gyrases, type-IA TOP1, TOP2, PolI, RNase HII, SSB, and LIG1. Several of these enzymes, including POP, TWINKLE, gyrases, type-IA TOP1, TOP2, LIG1, and PolI (or 5′–3′ exonuclease), were retained by all plants. In the early green lineage, WHY was obtained, and mitochondrial TWINKLE became dually localized in mitochondria and plastids. However, the dual-localization of TWINKLE resulted in the loss of DnaB and DnaG in the green lineage. After the divergence of Chlorophyta, the ancestor of land plants obtained the SSBs, OSB, and ODB. The acquisition of these SSBs, in addition to WHY, in the green lineage may have partially contributed to exploration of terrestrial habitats, as the high recombinant/repair activity of these enzymes would have potentially allowed the repair of DNA damaged by direct sunlight. The sets of RECA and WHY, and RECA and OSB were lost in Bryophyta and Pteridophyta, respectively, followed by the loss of RNase HII (homolog of *Cyanidioschyzon merolae* CMT626C) in Angiospermae. In red algae, most replication enzymes in the ancestor of photosynthetic eukaryotes are found in present-day species.

## CONCLUDING REMARKS

In the past decade, most enzymes related to plastid and mitochondrial DNA replication in plants and algae have been identified. These studies have revealed that the core enzymes and components involved replication are identical in the plastids and mitochondria of land plants. Because the nuclear genomes of green plants and algae encode these core replicative enzymes, such as POP, TWINKLE, gyrases, TOP1, TOP2, LIG1, and 5′–3′ exonuclease, which frequently contain putative dual-targeting sequences at the *N*-terminus (**Table 1**), it is presumed that the green lineage contains a similar set of plastid and mitochondrial enzymes. In contrast, SSBs and recombination-related enzymes are not universally conserved in the green lineage, suggesting that these enzymes are possibly susceptible to exchange or loss during evolution, leading to the acquisition or creation of species-specific enzymes. Unlike the green lineage, red algae contain different replicative protein profiles in plastids and mitochondria. Red algal plastids contain numerous replication proteins that originated from cyanobacteria (Moriyama et al., 2014), suggesting that the mechanism of genome replication in these plastids might be similar to that found in bacteria.

To date, a number of organelle-localized enzymes have been identified. However, biochemical data are lacking for the majority of organellar replication enzymes in plants. The role of an enzyme predicted by homology searches against known enzymes might differ from its actual function or properties. For example, soybean plastid DNA replication ODB shares homology with bacterial and *A. thaliana* SSB, but only binds to dsDNA of the *oriA* sequence in plastid DNA, and not to ssDNA (Lassen et al., 2011).

The regulatory mechanisms controlling the initiation of plant organellar genome replication and the number of organellar DNA copies remains to be explored. Recently, chloroplast DNA replication was shown to be regulated by the cellular redox state in the green alga *Chlamydomonas reinhardtii* (Kabeya and Miyagishima, 2013). Specifically, chloroplast DNA replication was activated and inactivated by the addition of reducing and oxidative agents, respectively, in both *in vivo* and *in vitro* assays. Light-dependent genome replication was also reported in cyanobacteria, in which DCMU [3-(3,4-dichlorophenyl)-1,1-dimethylurea], an inhibitor of electron transport between the PSII complex and plastoquinone pool, inhibits DNA replication initiation, and DBMIB (2,5-dibromo-3-isopropyl-6-methyl-p-benzoquinone), an inhibitor of electron transport between plastoquinone and cytochrome *b_6_f* complex, inhibits the initiation and elongation of replication (Watanabe et al., 2012; Ohbayashi et al., 2013). Thus, the light-mediated replication of plastid DNA in algae may have originated from cyanobacteria. However, organellar replication in land plants and multicellular plants appears to be regulated by other mechanisms. In land plants, the replication of organellar genomes is restricted to meristematic tissues, and is not associated with the cycle or organellar division (Hashimoto and Possingham, 1989; Fujie et al., 1993). These findings suggest that land plants have more complex regulatory mechanisms controlling the replication of organellar genomes than those operating in algae.

## Conflict of Interest Statement

The authors declare that the research was conducted in the absence of any commercial or financial relationships that could be construed as a potential conflict of interest.
